# Identification and Characterization of TF-lncRNA Regulatory Networks Involved in the Tumorigenesis and Development of Adamantinomatous Craniopharyngioma

**DOI:** 10.3389/fonc.2021.739714

**Published:** 2022-01-26

**Authors:** Dingkang Xu, Yufeng Guo, Shixiong Lei, Abao Guo, Dengpan Song, Qiang Gao, Shengqi Zhao, Kaiwen Yin, Qingjie Wei, Longxiao Zhang, Xiaoxuan Wang, Jie Wang, Qi Zhang, Fuyou Guo

**Affiliations:** ^1^ Department of Neurosurgery, The First Affiliated Hospital of Zhengzhou University, Zhengzhou, China; ^2^ Department of Urology, First Affiliated Hospital of Zhengzhou University, Zhengzhou, China; ^3^ Department of Pharmacology, School of Pharmaceutical Sciences, Zhengzhou University, China, Zhengzhou, China; ^4^ State Key Laboratory of Esophageal Cancer Prevention & Treatment, Zhengzhou University, Zhengzhou, China

**Keywords:** craniopharyngiomas, LncRNA, transcription factors, RNA-seq, integrated algorithm

## Abstract

Craniopharyngiomas (CPs) are rare tumors arising from the sellar region. Although the best outcome for patients with one subtype, adamantinomatous craniopharyngioma (ACP), is obtained by gross total resection, little is known about the roles of long noncoding RNAs (lncRNAs) and transcription factors (TFs) in ACP tumorigenesis. In total, 12 human ACP and 5 control samples were subjected to transcriptome-level sequencing. We built an integrated algorithm for identifying lncRNAs and TFs regulating the CP-related pathway. Furthermore, ChIP-Seq datasets with binding domain information were used to further verify and identify TF-lncRNA correlations. RT–PCR and immunohistochemistry staining were performed to validate the potential targets. Five pathways associated with ACP were identified and defined by an extensive literature search. Based on the specific pathways and the whole gene expression profile, 266 ACP-related lncRNAs and 39 TFs were identified by our integrating algorithm. Comprehensive analysis of the ChIP-Seq datasets revealed that 29 TFs were targeted by 12000 lncRNAs in a wide range of tissues, including 161 ACP-related lncRNAs that were identified by the computational method. These 29 TFs and 161 lncRNAs, constituting 1004 TF-lncRNA pairs, were shown to potentially regulate different ACP-related pathways. A total of 232 TF-lncRNA networks were consequently established based on differential gene expression. Validation by RT–PCR and immunohistochemistry staining revealed positive expression of the ACP-related TFs E2F2 and KLF5 in ACP. Moreover, the expression of the lncRNA RP11-360P21.2 was shown to be upregulated in ACP tissues. In this study, we introduced an integrated algorithm for identifying lncRNAs and TFs regulating the ACP-related pathway. This is the first comprehensive study to systematically investigate the potential TF and lncRNA regulatory network in ACP. The resulting data serve as a valuable resource for understanding the mechanisms underlying ACP-related lncRNAs and TFs.

## Introduction

Craniopharyngiomas (CPs) are benign suprasellar tumors and account for 2-4% of all intracranial tumors (1, 2). Despite their histological classification as WHO I tumors, CPs remain challenging to treat *via* total resection and postoperative management due to their specialized location and biological behavior, leading to increased mortality and poor functional results, similar to those of patients with severe endocrine disorders.

There are two types of CPs, namely, adamantinomatous craniopharyngiomas (ACPs) and papillary craniopharyngiomas (PCPs). ACPs exhibit a bimodal incidence, peaking in both childhood and at 45-60 years (1–3). The important genomic characteristics of ACPs are somatic mutations of CTNNB1, which encodes β-catenin, as revealed by widespread whole-exon sequencing, while PCPs are driven by mutation of p. BRAF-V600E (4). CPs harboring histological features of both ACP and PCP are extremely rare (5). Recently, multiple studies have provided new insight into the tumorigenesis of ACP and possible therapeutic targets. Mutation in exon 3 of CTNNB1, leading to overactivation of the Wnt/β-catenin signaling pathway, is considered the main oncogenic driver of ACP tumorigenesis. Moreover, other pathways, such as ERBB2 and SHH signaling, have also been shown to be related to tumor growth and proliferation (6–8). Gaston et al. constructed an ACP embryonic and inducible model to further confirm that cells in the β-catenin-accumulating cluster promote tumor growth by regulating paracrine cells with a series of related proteins, including bone morphogenic proteins (BMPs) and fibroblast growth factors (FGFs) (9, 10). Transcriptome sequencing of murine ACP models and human tumor samples using laser capture microdissection has revealed overactivated MAPK/ERK pathways in tumor components, implying that MEK inhibitors could be developed as a potential treatment (11). Grob et al. reported high expression of IL-6 in ACPs, and current therapies include IL-6 inhibitors and have shown satisfactory results in patients with cystic ACP (12). The differential genetic backgrounds and epigenetic factors of ACP and PCP lead to differences in targeted therapies. Therefore, further studies at the transcription level are essential for elucidating the potential molecular mechanism of ACP.

Long noncoding RNAs (lncRNAs) are a type of RNA exceeding 200 nucleotides in length that are not translated into proteins. Mounting evidence suggests that lncRNAs impact numerous biological processes, such as cell proliferation, invasion, differentiation, apoptosis and metastasis (13). Similarly, transcription factors (TFs) are thought to modulate the expression of lncRNAs, thereby mediating the expression of downstream molecules and promoting cancer development (14). Recently, Li et al. explored the regulatory roles of lncRNAs in different immune-related pathways in tumors using the GSEA method (15). Because ACP, which is considered a benign tumor, carries a low rate of somatic mutations, mounting evidence suggests that the TGF-β, ERBB2 and SHH signaling pathways are involved in tumor formation in an autocrine or paracrine manner. However, only a few studies focusing on the noncoding transcriptome of ACP tumors have been reported (16, 17).

To systematically identify the regulatory networks that are potentially associated with ACP, we utilized an integrated algorithm to specifically explore the TFs and lncRNAs regulating ACP. This is the first study to investigate TFs and lncRNAs that affect the biological behavior of ACP. In addition, we further constructed TF-lncRNA pairs, which provided new insights into the mechanism underlying ACP, and we demonstrated that the expression levels of E2F2, KLF5 and RP11-360P21.2 were significantly upregulated in ACP tissues. In conclusion, this comprehensive study on TFs and lncRNAs was performed to investigate the pathogenesis and underlying mechanism of ACP development.

## Methods and Materials

### Clinical Samples

In total, 12 ACP samples and 5 normal brain tissue samples were collected from patients at the First Affiliated Hospital of Zhengzhou University, PR China. All procedures were approved by the Ethics Committee for Human Experiments of Zhengzhou University. Informed consent was obtained, and the University Review Board approved this study, which was conducted in accordance with the Helsinki Declaration. In this study, 12 ACP samples and 5 normal tissues were subjected to high-throughput RNA sequencing (RNA-seq) analysis, and 14 samples were subjected to further immunohistochemical staining. Five normal brain tissues were obtained from patients undergoing brain tissue resection due to severe traumatic intracerebral injury, and the ages and sexes of the patients in the two groups did not significantly differ.

### Whole RNA Sequencing

RNA isolation and quantification were conducted according to previous reports (18). Briefly, a total of 3 µg of RNA per sample was used as input material for the RNA sample preparations. First, ribosomal RNA was removed using an Epicenter Ribo-ZeroTM rRNA Removal Kit (Epicenter, USA), and rRNA-free residues were removed by ethanol precipitation. Subsequently, sequencing libraries were generated using rRNA-depleted RNA with an NEBNext UltraTM Directional RNA Library Prep Kit for Illumina (NEB, USA) in accordance with the manufacturer’s recommendations. The total RNA was sequenced on a HiSeq 4000 platform (Illumina, USA) at a read length of 2 × 150 bp. Then, the limma R package was used to conduct differential gene expression analysis. The adjusted P values were analyzed to correct for false positive results in the datasets. An adjusted P < 0.05 and a log (fold change) >1 or log (fold change)< −1 were defined as the thresholds for screening differential mRNA expression.

### Identification of lncRNAs and TFs in ACP-Related Pathways

Because the Wnt/β-catenin, SHH, TGF-β, ERK1/ERK2, MAPK and ERBB2 pathways are involved in the development of CP (6–8, 10), we defined the above pathways as ACP-related pathways. To identify potential lncRNAs in ACP-related pathways, we used a calculation method involving the integration of lncRNAs and whole gene expression data. We used the method described by Li et al. with some modifications to identify TFs and lncRNAs modulating ACP (15). In brief, all genes were ranked based on their correlation with lncRNA/TF expression. To determine whether the gene sets were enriched in ACP-related pathways, the ranked genes and related pathways were calculated. The lncRNA relative enrichment score (lncRES) of each lncRNA pathway was computed, and pairs with significant values were identified. For each lncRNA or TF, we computed its activity in ACP pathways (lncRES/or TFRES) based on modified gene set enrichment analysis (GSEA).

Based on the correlation of the expression of coding genes with lncRNAs/TFs, we ranked these lncRNAs/TFs in order. Each lncRNA/or TF i and coding gene j were defined as follows: lncRNA(i) = (lncRNA1, lncRNA2, lncRNAi, …, lncRNAm) and gene(j) = (gene1, gene2, gene3, …genej,… genem).


pcorValue(ij)=pearson(ij)

For each lncRNA-gene pair, the rank score (RS) was calculated as follows:


$$RS(\text{ij})=\log_{10}(p(ij))*pcorValue(ij)$$

where p(ij) is the Pearson’s (ij) P value.

Genes were ranked according to the RES values and subjected to enrichment analysis. To analyze the regulatory network(s) between lncRNAs and ACP-related pathways, we mapped the genes to the rank list according to the principles of GSEA. Then, the enrichment score (ES) based on the GSEA was calculated. ES_ik_ was defined as the ES between the lncRNA i and ACP-related pathway k. Finally, we integrated the P value and the ES into lncRES values as follows.


$$LncRES\left(i,k\right)=\{\begin{array}{c} 1-2p;if\;ES\left(ik\right)>0\\ 2p-1;if\;ES\left(ik\right)<0 \end{array}$$

Consequently, the lncRESs ranged from -1 to 1, and an absolute lncRES >0.995 and a false discovery rate (FDR) <0.05 were considered to indicate significant lncRNA-pathway pairs. The TF pathways were constructed with the same method described above. To acquire a relatively reasonable number of TFs, TFs with a RES >0.990 were considered significant.

### Identification of TF-lncRNA Regulatory Interactions

To identify potential TF-lncRNA relationships, we downloaded a large number of ChIP-seq peak datasets of TFs from the ChIPBase database (http://rna.sysu.edu.cn/chipbase/) (14, 19). In ChIPBase v2.0, 10200 peak datasets generated from ChIP-seq, ChIP-exo and MNChIP-seq datasets were curated from the NCBI GEO database, ENCODE project, modENCODE project and NIH Roadmap Epigenomics project. We extracted the peak data of TFs from all lncRNA datasets and validated the peak information regarding lncRNA/TF regulators associated with ACP-related pathways. Specific TF-lncRNA pairs were established when a peak signal was observed between TFs and lncRNAs. A total of 1004 potential TF-lncRNA interactions among 29 TFs and 161 lncRNAs were generated based on the conserved TF binding sites and ChIP-Seq dataset. Consequently, we selected the significant TFs and lncRNAs from the differentially expressed genes, and a total of 232 TF-lncRNA pairs were constructed. The “OmicCircos” package (R) was used to visualize the severity of the gradients of the normalized fold changes in the expression levels of the top lncRNAs and TFs across all studies on ACP (20).

### Quantitative Reverse Transcription PCR Analysis (RT–PCR)

The real-time RT–PCR method used was described in our previous study (21). Briefly, oligonucleotide primers and TaqMan probes for KLF5 and E2F2 were designed based on sequences available in the GenBank database. The PCR primer sequences designed for amplification of the target molecules are listed in the **Supplemental Table**. The conditions for real-time RT–PCR were as follows: preheating at 94°C for 2 min, followed by 35 cycles of 94°C for 20 s, 60°C for 40 s, and 72°C for 30 s. The quantity of the β-actin gene product, a representative housekeeping gene, was equivalent in all the samples. Relative changes in gene expression were quantified using the likelihood method (2^−ΔΔCt^ method). The normalized value for each target cDNA reflected the expression level of the corresponding gene.

### Immunohistochemistry Staining

The immunohistochemistry method used in the present study has been previously described (18, 21). Briefly, sections were incubated overnight at 4°C with the following primary antibodies: rabbit anti-KLF5 (1:1000, Proteintech, China) and rabbit anti-E2F2 (1:1000, Proteintech, China). After washing, a goat anti-rabbit antibody (1:200, Servicebio, China) was added and incubated at room temperature.

### Statistical Analysis

Genes were considered differentially expressed if they had a fold change ≥ 2 and a P value < 0.05. Parametric data are presented as the mean ± standard deviation. The mean integrated optical density (IOD) was used to compare the immunohistochemical staining differences between the two groups. Differences between two groups were evaluated with the two-tailed Student’s t test. All analyses were performed with R software (Version 3.6.3). P<0.05 was considered statistically significant.

## Results

### Patients Characteristics and Grouping

In total, 12 primary ACP samples and 5 control brain tissues were subjected to RNA-seq analysis. The ACP group included 5 males and 7 females, 4 of whom were children and 8 were adults (5,7,10,13 years old vs 22,33,51,54,56,56,57,58 years old). The control group included 3 males and 2 females, with an average age of 40 years. There was no significant difference between the ACP and control groups.

### Identification of lncRNAs/TFs in ACP-Related Pathways

**Figure 1:**
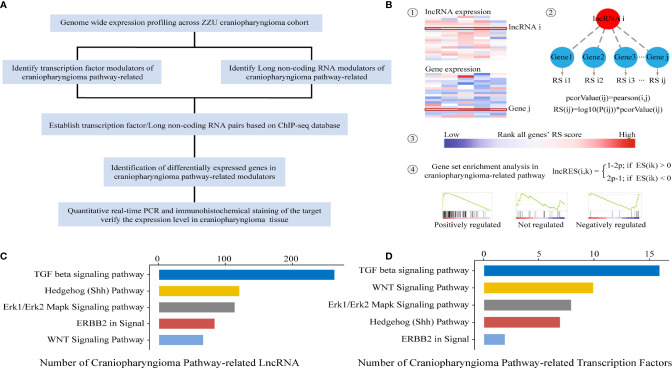
Schematic of data generation and analysis **(A)**. Workflow patterns of lncRNA-pathway and TF-pathway network construction **(B)**. ACP pathway-related TFs and lncRNAs with significant RESs **(C, D)**.

Previous studies have indicated that the Wnt/β-catenin, SHH, TGF-β, Erk1/Erk2 MAPK and ERBB2 pathways play significant roles in the occurrence and development of CP. In the present study, the identification of lncRNAs/TFs was based on ACP-related pathways. To identify potential lncRNAs and TFs correlated with ACP-related pathways, an integrated algorithm was used to construct TF- and lncRNA-pathway networks (**Figure 1A**). First, the whole gene expression profiles of mRNAs and lncRNAs were obtained. Then, we calculated and ranked the RESs of genes for each lncRNA. Third, the activity of each lncRNA in the ACP-related pathway was calculated based on GSEA, and the P values were converted into lncRES values (**Figure 1B**). A lncRES >0.995 and an FDR <0.05 were considered significant. According to the relevant lncRNA-pathway pairs, the lncRNA-related pathways were in the following descending order: TGF-β, SHH, Erk1/Erk2 MAPK, ERBB2 and Wnt/β-catenin (**Figure 1C**). In addition, the TF-related pathways were in the following descending order: TGF-β, Wnt/β-catenin, Erk1/Erk2 MAPK, Shh, and ERBB2 pathways (**Figure 1D**).

**Figure 2:**
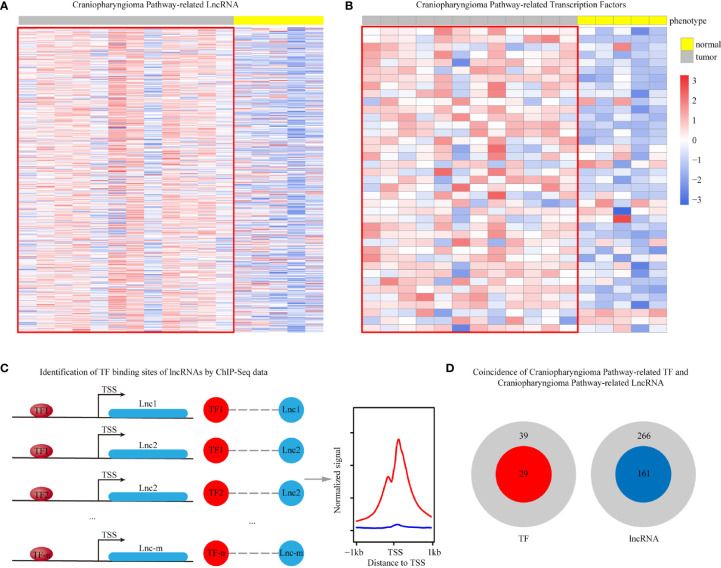
Heatmap showing the differentially expressed ACP pathway-related lncRNAs **(A)** and TFs **(B)**. The schematic diagram shows the binding process of TFs and lncRNAs (**C**, red represents TFs and blue represents lncRNAs). In total, 266 lncRNAs and 39 TFs were identified. Of these, 161 lncRNAs and 29 TFs with binding domain information were ultimately verified through ChIP datasets (**D**, red represents TFs and blue represents lncRNAs).

**Figure 3:**
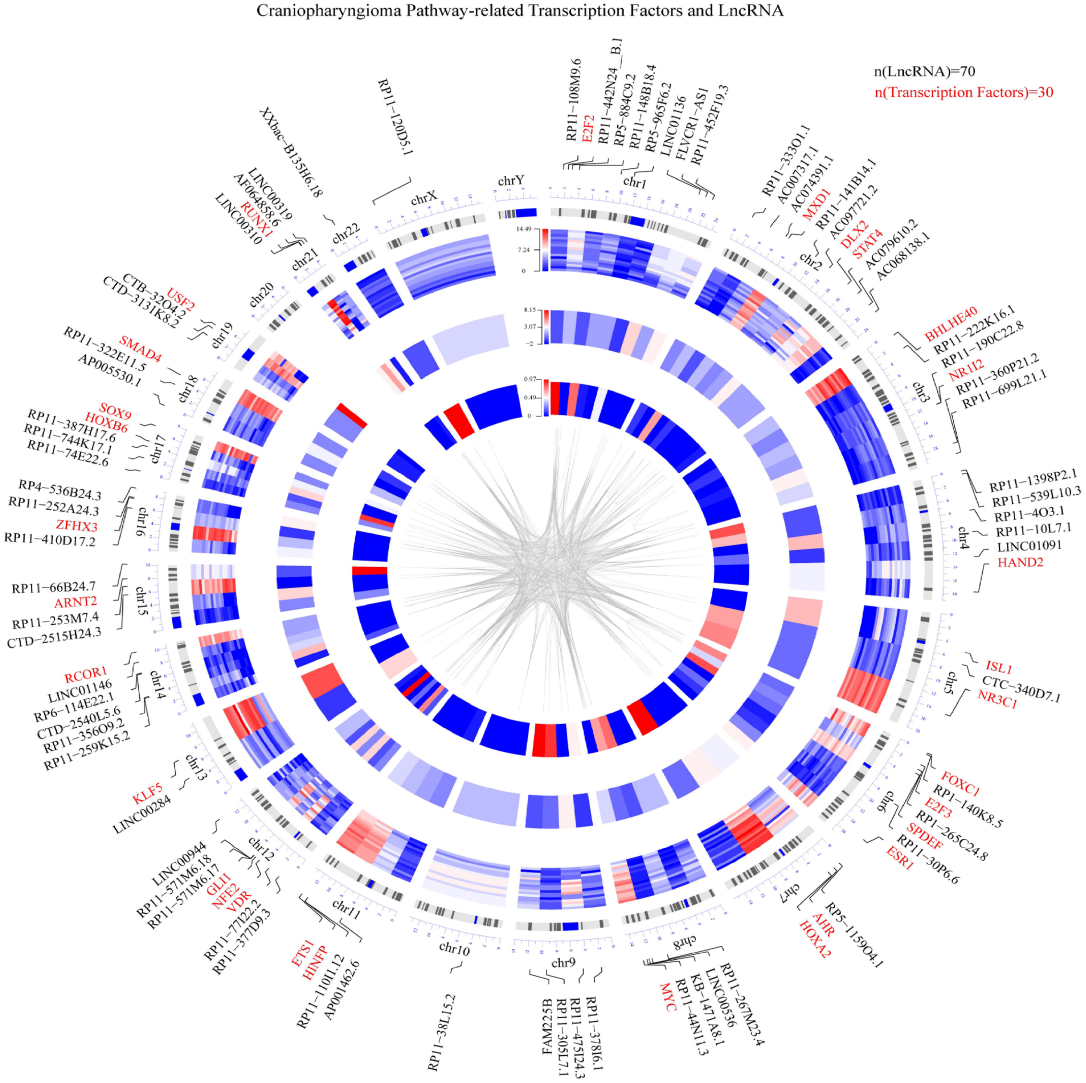
Circular visualization of the chromosomal positions, differential expression levels and P values of the top 30 TFs and top 70 lncRNAs. The concentric circles representing different microarray datasets radiate outwards from the middle of the circle, with the P values shown in the innermost group, the fold changes shown in the middle group and the differential expression of each gene shown in the outermost group. The lines from each gene symbol represent its specific chromosomal location. A weighted heatmap image was constructed according to the log2-fold changes in gene expression regardless of significance. The gray lines in the center of the circle indicate a specific interaction between the TF and lncRNA based on the ChIP datasets (red represents upregulation, and blue represents downregulation).

Based on the integrated algorithm, we identified TFs and lncRNAs of the ACP-related pathway, and the expression of TFs/lncRNAs was significantly upregulated in ACP tissues (**Figures 2A, B**). First, 39 TFs and 266 lncRNAs were identified, and 161 of these overlapped with ChIP-Seq datasets and ACP profiles (Supplemental material, See TFRES and LncRES). Then, significant genes among the 5 signaling pathways were identified. The top 15 TF-pathway and lncRNA-pathway pairs are listed in **Table 1**. Finally, 1004 TF-lncRNA interactions among 29 TF pathways and 161 lncRNA pathways were constructed using the ChIP-Seq datasets (Supplemental material, see TF-lncRNA) (**Figures 2C, D**). In addition, the positions of the top 30 TFs and the top 70 lncRNAs on the chromosome and their expression patterns are shown in **Figure 3**.

**Table 1:** Top 15 TFs and lncRNAs of the ACP-related pathway.

	Gene	RES	FC	P	Pathway
TFs	KLF5	0.997719498	6.309701298	5.78715E-06	WNT signaling pathway
	RUNX1	0.99252802	4.835681265	5.29484E-07	TGF-β signaling pathway
	ISL1	0.997392438	4.460237557	4.11582E-05	Erk1/Erk2 Mapk signaling pathway
	GLI1	0.997775306	4.077460079	0.000782605	Hedgehog (Shh) pathway
	VDR	0.992217899	3.747748682	0.00040768	TGF-β signaling pathway
	DLX2	0.997329773	3.268986894	0.023339632	WNT signaling pathway
	AHR	0.994962217	3.267678968	1.86192E-06	TGF-β signaling pathway
	ESR1	-0.991404011	3.085325484	0.001337029	Erk1/Erk2 Mapk signaling pathway
	HAND2	-0.99488491	2.868591174	0.003974244	TGF-β signaling pathway
	HOXB6	-0.992471769	2.748524623	0.001430767	WNT signaling pathway
	MYC	0.994100295	2.410371459	0.000783643	Erk1/Erk2 Mapk signaling pathway
	ETS1	-0.99375	2.16777249	5.19216E-05	Hedgehog (Shh) pathway
	BHLHE40	0.997329773	2.012126829	0.001622299	TGF-β signaling pathway
	ARNT2	-1	-1.996219981	0.006013635	WNT signaling pathway
	E2F2	0.992277992	1.861109149	0.00236967	Hedgehog (Shh) pathway
LncRNAs	ZNF888	0.995979899	8.14568238	8.8122E-08	WNT signaling pathway
	RP11-356O9.2	-0.995584989	4.575635245	8.83516E-09	TGF-β signaling pathway
	RP11-65L19.4	-0.996779388	4.30060785	5.08636E-09	ERBB2 signaling pathway
	LINC00426	-0.996357013	4.274391208	5.22651E-09	Hedgehog (Shh) pathway
	RP11-373D23.3	0.997641509	4.248671731	6.69968E-07	TGF-β signaling pathway
	RP11-55L3.1	-0.997903564	3.846392873	3.67095E-07	WNT signaling pathway
	RP4-781K5.6	-0.997412678	3.673846446	1.65208E-06	ERBB2 signaling pathway
	CTB-1I21.1	-0.995085995	3.474261135	1.29922E-06	ERBB2 signaling pathway
	CTC-490G23.2	-0.997663551	3.437881709	1.65967E-06	WNT signaling pathway
	RP11-475I24.3	0.997894737	3.437581722	0.000789382	Hedgehog (Shh) pathway
	AC012363.13	-0.996621622	3.129375139	9.57969E-08	TGF-β signaling pathway
	RP11-452F19.3	0.997742664	3.117381748	5.30894E-06	Erk1/Erk2 Mapk signaling pathway
	CTC-529P8.1	-0.997572816	-3.107091438	0.000117385	ERBB2 signaling pathway
	CTC-529P8.1	-0.995203837	-3.107091438	0.000117385	Erk1/Erk2 Mapk signaling pathway
	RP11-360P21.2	-0.996632997	3.080472936	1.7834E-06	TGF-β signaling pathway

### Gene Expression and Construction of TF-lncRNA Pairs

Differential gene expression analysis revealed a total of 293 RNAs that were differentially expressed. Of these RNAs, the expression levels of 59 RNAs were decreased, while the expression levels of the remaining RNAs were significantly increased (Supplemental material). The differential expression and Gene Ontology (GO) analysis were investigated between pediatric and adults (Supplemental material, see pediatric vs adults). The top 3 related biological processes (BP) were production of molecular mediator of immune response, humoral immune response and immune response-activating cell surface receptor signaling pathway (**Supplemental Figure**).

To explore the TF-lncRNA pairs potentially playing roles in the occurrence and development of ACP, we established a TF-lncRNA network based on the ChIP-seq database. In summary, a total of 232 TF-lncRNA pairs were finally established after matching significant RNAs in the profile (Supplemental material, see TF-lncRNA pairs). For example, the STAT4 TF targeted 10 lncRNAs, and KLF5 was correlated with 25 KLF5-lncRNA pairs. Of note, a pair could affect multiple pathways, and a pathway could also be affected by multiple pairs, indicating that the interaction was not one-to-one.

**Figure 4:**
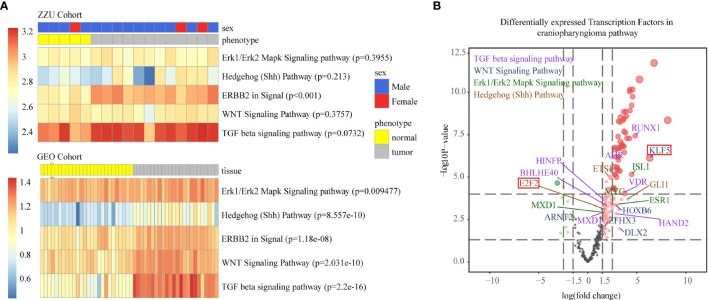
Single-sample gene set enrichment analysis (ssGSEA) of the ZZU and GEO cohorts **(A)**. The volcano map shows the fold changes and P values corresponding to the differentially expressed TFs among different TF-pathway pairs. The volcano plot shows the differences in mean gene expression between the ACP and control samples on the x-axis and the corresponding –log10 transformed p values on the y-axis. The different colors represent the various related pathways, and the colors represent the pathway with the highest RESs (**B**, purple represents the TGF-β pathway, blue represents the Wnt pathway, green represents the Erk1/Erk2 pathway and brown represents the Shh pathway).

To further verify the reliability of the TF and lncRNA pathways we identified, we matched the data obtained from our center with those obtained from the GSE94349 dataset (**Figure 4A**) (22). Only the GEO cohort was used to quantify pathway activity. Although not completely significant in the ZZU dataset, which may have been due to data error and the small number of samples, all the data in the Gene Expression Omnibus (GEO) cohort were significantly correlated with the five pathways. The final TFs were exported based on the whole gene expression profile and significant differentially expressed genes (**Figure 4B**).

### Confirmation of the Expression Changes in ACP-Related Genes

**Figure 5:**
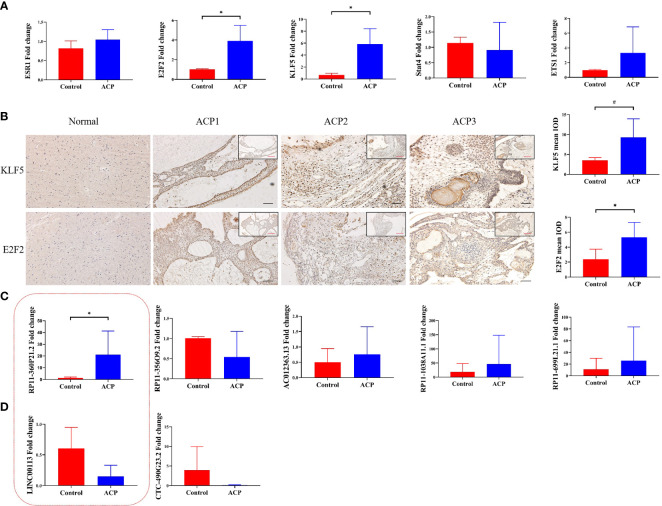
RT–PCR analysis of ESR1, E2F2, KLF5, STAT4 and ETS1 at the transcript level. The expression of E2F2 and KLF5 was markedly increased in ACP tissues compared with normal brain tissues (**A**, n=5, * indicates p < 0.05). Representative immunochemistry images of KLF5 and E2F2 in ACP. Moderate staining of KLF5 and E2F2 was observed in normal tissues (**B**, n=14, * indicates p < 0.05, # indicates p < 0.01). RT–PCR analysis revealed the expression of related lncRNAs binding to KLF5- **(C)** and E2F2-lncRNA pairs **(D)**. The red box represents lncRNAs that bind to both KLF5 and E2F2. The results indicated increased expression of RP11-360P21.2 (n=8, * indicates p < 0.05, # indicates p < 0.01).

To confirm the real expression of TFs/lncRNAs in ACPs, highly significantly expressed TFs/lncRNAs of interest were detected, including 5 TFs (KLF5, E2F2, STAT4, ETS1 and ESR1) and 7 lncRNAs. qRT–PCR was performed, revealing that the expression levels of KLF5 and E2F2 were increased in the ACP group compared with the control group (p<0.05, **Figure 5A**). The expression levels of STAT4, ETS1, or ESR1 did not significantly differ between the tumor and control groups. Furthermore, the ACP samples were subjected to immunohistochemistry analysis, which demonstrated strong staining of KLF5 and E2F2 in the whole tumor tissues, especially in the cystic wall (**Figure 5B**). We further screened the top 5 lncRNA pairs with the top FC values among the TF-lncRNA pairs (**Table 2**). The expression of RP11-360P21.2 was significantly increased in ACP (**Figures 5C, D**). Thus, based on the PCR, immunohistochemistry, and integrated algorithm results, we established the KLF5-RP11-360P21.2 and E2F2-RP11-360P21.2 pairs in ACP.

**Table 2:** Top5 TF-lncRNA pairs.

TF-lncRNA pairs		
Pair	logFC	adj. P. value
KLF5-RP11-360P21.2	3.080472936	1.78E-06
KLF5-RP11-699L21.1	2.287542186	6.55318E-05
KLF5-LINC00113	2.055964371	0.000597201
KLF5-RP11-267M23.4	1.676662109	0.005338561
KLF5-RP11-110I1.12	1.122476444	0.016541618
E2F2-CTC-490G23.2	3.437881709	1.65967E-06
E2F2-RP11-360P21.2	3.080472936	1.7834E-06
E2F2-RP11-699L21.1	2.287542186	6.55318E-05
E2F2-LINC00113	2.055964371	0.000597201
E2F2-RP11-267M23.4	1.676662109	0.005338561
ETS1-RP11-360P21.2	3.080472936	1.7834E-06
ETS1-RP11-699L21.1	2.287542186	6.55318E-05
ETS1-RP11-267M23.4	1.676662109	0.005338561
ETS1-RP1-265C24.8	-1.279511209	0.021678162
ETS1-RP11-110I1.12	1.122476444	0.016541618
STAT4-RP11-699L21.1	2.287542186	6.55318E-05
STAT4-RP11-267M23.4	1.676662109	0.005338561
STAT4-RP1-265C24.8	-1.279511209	0.021678162
ESR1-ZNF888	8.14568238	8.8122E-08
ESR1-RP11-356O9.2	4.575635245	8.83516E-09
ESR1-LINC00426	4.274391208	5.22651E-09
ESR1-RP11-55L3.1	3.846392873	3.67095E-07
ESR1-CTB-1I21.1	3.474261135	1.29922E-06

## Discussion

In recent years, due to the development of high-throughput technology, research on CP has been transferred from the genome level to the transcriptome level, but research at the nontranscriptome RNA level is still lacking in this field. Mounting evidence suggests that lncRNAs are important modulators of tumorigenesis (23, 24), but no research on TF/lncRNA regulation in ACP has been performed. In this study, we established TF-lncRNA networks by using an integrated algorithm based on high-throughput sequencing data of ACP. We first summarized the ACP-related pathways and identified the pathway-related TFs and lncRNAs based on whole gene expression. Furthermore, we validated the TF-lncRNAs pairs based on exact binding domain information verified by ChIP-Seq data and found that RP11-360P21.2 was upregulated in ACP. Finally, the TF-lncRNA pairs were obtained by matching the significant differentially expressed genes. Increased KLF5 and E2F2 expression levels were observed in tumor tissues, especially in the palisading epithelium and the cyst wall. Therefore, the binding sites of KLF5-RP11-360P21.2 and E2F2-RP11-360P21.2 may become regulatory networks to mediate the expression of downstream genes playing roles in ACP development. To the best of our knowledge, this is the first study focusing on lncRNAs and TFs in ACP, and the results potentially provide pathological insight into the mechanism underlying the unique growth pattern of ACP.

The pathogenesis of CP is complex and involves not only driver mutations but also coding and noncoding RNAs. ACPs are a series of tumors that have low incidence rates, which limits our knowledge of these tumors at the transcriptome level, especially at the noncoding RNA level. Although our knowledge about ACP has increased, the specific regulatory network of the underlying mechanism is still unclear. Increasing evidence suggests that TFs and lncRNAs play key roles in the progression of multiple cancers (25, 26). Therefore, the identification of TFs and lncRNAs is critical for the construction of TF-lncRNA pairs. Although several studies on CPs utilized RNA-seq analysis, most of them were limited to the expression of a few genes and lack an overall profile. Previous studies have revealed that SOX2+ pituitary stem cells may cause tumorigenesis in a paracrine manner based on the ACP mouse model (9, 10, 23). Evidence indicates that the tumor as a whole is complex and that one or few molecules might not be sufficient to explore the nature of the tumor. Considering the low genomic mutation rate in ACP, determining which factors among bulk gene sets that play essential roles in its biological process is difficult. Thus, we developed this method as a different validation process, and the results indicated its potential applications for prioritizing ACP-related pathway lncRNAs and TFs. The TF, lncRNA and TF-lncRNA networks have already provided novel biological insight into ACP. For instance, ESR1 yielded a high TFRES value in the ERK1/2 MAPK pathway (**Table 1**), indicating that it plays a preferential regulatory role in this pathway in ACP. ESR1, which is responsible for the maintenance of bone integrity, may play a key role in ACP osteogenesis. Generally, the increased activation of the ERK1/2 MAPK pathway is accompanied by the repression of ESR1 expression (27); however, the development of ACP involves the participation of both the ERK1/2 MAPK pathway and ESR1 (**Table 1**). This phenomenon may be attributed to the differential transcriptional backgrounds of different cell types. In addition, BMP2 was mainly expressed in the stellate reticulum and whorl-like array, while strong ERK1/2 staining was observed in the palisading epithelium. These observations suggest that these TFs and lncRNAs will be beneficial for prioritizing ACP-related lncRNAs and TFs, leading to the identification of several novel genes as potential targets in ACP. Notably, bulk RNA sequencing might allow us to ignore some specific cellular components, and more advanced sequencing such as single-cell sequencing should serve as a new tool to better understand the tissue components in craniopharyngioma in the future.

Noncoding RNAs are emerging as critical factors involved in posttranscriptional regulation, but only a few studies have focused on this field in ACP (16, 17). ACP exhibits a distinct pathological structure with differential expression levels. The activation of MAPK/ERK signals occurs mainly in the palisading epithelium and reactive glial tissues, whereas BMP signaling-activated cells lie within and adjacent to β-catenin cluster cells (10). Based on previous reports, we focused more on TFs related to the processes of epidermal development, keratinocyte differentiation and odontogenesis (28). In this study, two TFs, E2F2 and KLF5, were confirmed to be differentially expressed. KLF5 encodes a member of the Krüppel-like factor zinc finger protein family and is involved in a variety of physiological processes, including proliferation, differentiation and embryogenesis. Ng et al. reported that KLF5 promotes tumor epithelial development in patients with Barrett’s esophagus (BE) and esophageal adenocarcinoma (29). Our study revealed that KLF5 may also play the same biological role in the development of ACP. KLF5 promotes the nuclear localization and transcriptional activity of β-catenin through a physical interaction (30), revealing its crucial role in the Wnt/β-catenin pathway. In addition, KLF5 binds to the RUNX2 promoter to mediate vascular smooth muscle cell calcification (31). E2F2 is a member of the E2F TF family and is mainly involved in regulating the cell cycle. Increased E2F2 expression is potentially predictive of a poor prognosis in hepatocellular carcinoma and of inflammatory cytokine upregulation (32, 33). In addition, several striking features were observed in the present study. Although the two TFs detected by RNA-seq are highly expressed in CPs, their expression levels differ depending on their pathological features. The expression of E2F2 and KLF5 was more prominent at the edge of the tumor cystic wall. Because the CP cyst is a unique feature, the cystic fluid may be a microenvironment that promotes CP growth. Previous studies have reported that inflammasomes are activated in ACP and that cholesterol crystals are potential activators, providing insight into the generation of cystic fluid and the growth pattern of ACPs (11, 23). The occurrence of CP may be related to several inflammatory factors, such as the IL-1- and IL-6-mediated senescence-associated secretory phenotype (SASP) phenotype (23). High E2F2 expression and E2F2-RP11-360P21.2 binding may mediate downstream inflammatory factors and thus mediate the growth of CP. Due to the limitation of TF-lncRNA domain information in the CHIP-seq database, the regulatory network in CPs can be further developed using the integrated algorithm with newly acquired information in the future.

This study had several limitations, including an in-depth study on differential expressions between adults and pediatric groups, a shortage of link between pathways and phenotypes such as epithelial-mesenchymal transition (34, 35). In addition, the expression of more TFs and lncRNAs needs to be validated in a larger independent cohort. Future studies need to further clarify the role of the KLF5-RP11-360P21.2 and E2F2-RP11-360P21.2 regulatory networks in the development of ACP.

## Conclusion

In conclusion, this study identified the potential lncRNAs and TFs in ACP and established a TF-lncRNA regulatory network in ACP at the posttranscriptional level through RNA sequencing. The present study further clarified the regulation of ACPs at the posttranscriptional level. Targeting the KLF5-RP11-360P21.2 and E2F2-RP11-360P21.2 network may serve as a novel therapeutic strategy for ACP in the future. Together, these results suggest that the identification of critical lncRNAs/TFs involved in ACP can serve as a valuable resource in the development of precision medicine.

## Data Availability Statement

The datasets generated for this study can be found in The National Omics Data Encyclopedia (NODE). Datalink: https://www.biosino.org/node (Accession No: OER236537).

## Ethics Statement

The studies involving human participants were reviewed and approved by The Ethics Committee for Human Experiments of Zhengzhou University. Written informed consent to participate in this study was provided by the participants’ legal guardian/next of kin.

## Author Contributions

FG, DX, and YG designed the research. DX and YG performed the research and data analysis. DX, SL, QG, DS, SZ, KY, MZ, and LZ performed the basic studies. XW and JW collected the data. DX and YG wrote the paper. FG and QZ critically revised the paper. All authors contributed to the article and approved the submitted version.

## Funding

This work was supported by grants from the National Natural Science Foundation of China (U1204807), the Science and Technology Department of Henan Province (192102310113), the Medical Science and Technique Foundation of Henan Province (SB201901007) and the Educational Department of Henan Province (19B320017).

## Conflict of Interest

The authors declare that the research was conducted in the absence of any commercial or financial relationships that could be construed as a potential conflict of interest.

## Publisher’s Note

All claims expressed in this article are solely those of the authors and do not necessarily represent those of their affiliated organizations, or those of the publisher, the editors and the reviewers. Any product that may be evaluated in this article, or claim that may be made by its manufacturer, is not guaranteed or endorsed by the publisher.

## Supplementary Material

Supplementary Figure Enrichment analysis of differential genes between pediatric and adults. The size of the dot indicates the number of target genes, and the color represents the p value.
