# Supplementary figures and images for: Identification and Characterization of TF-lncRNA Regulatory Networks Involved in the Tumorigenesis and Development of Adamantinomatous Craniopharyngioma
Source: Front Oncol. 2022 Jan 26;11:739714. doi: 10.3389/fonc.2021.739714 (PMC8827039; doi:10.3389/fonc.2021.739714)

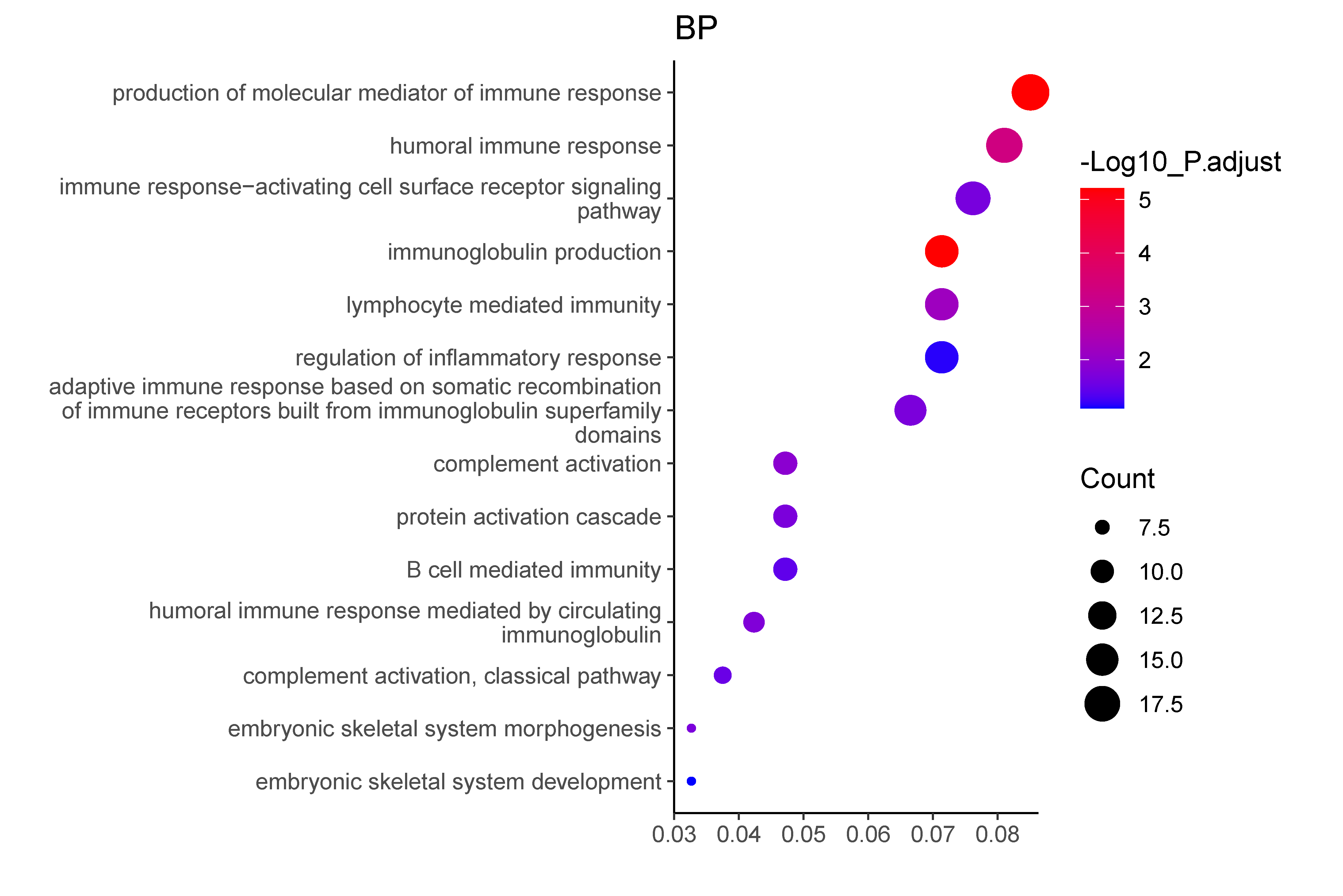

Supplement: Supplementary Figure — Enrichment analysis of differential genes between pediatric and adults. The size of the dot indicates the number of target genes, and the color represents the p value. [file Image_1.tif]
