# Supplementary material for: Identification and Characterization of TF-lncRNA Regulatory Networks Involved in the Tumorigenesis and Development of Adamantinomatous Craniopharyngioma
Source: Front Oncol. 2022 Jan 26;11:739714. doi: 10.3389/fonc.2021.739714 (PMC8827039; doi:10.3389/fonc.2021.739714)
Supplement: Supplementary file 3 [file Table_1.docx]

Table 1: Primer sequences used for qRT-PCR

| Name | Sequences(5'to 3') |
| --- | --- |
| ETS1 | Forward:GATAGTTGTGATCGCCTCACC |
|  | Reverse:GTCCTCTGAGTCGAAGCTGTC |
| ESR1 | Forward:GAAAGGTGGGATACGAAAAGACC |
|  | Reverse:GCTGTTCTTCTTAGAGCGTTTGA |
| E2F2 | Forward:CGTCCCTGAGTTCCCAACC |
|  | Reverse:GCGAAGTGTCATACCGAGTCTT |
| KLF5 | Forward:TCAGTCGTAGACCAGTTCTTCA |
|  | Reverse:CTGGGATTTGTAGAGGCCAGT |
| STAT4 | Forward:TGTTGGCCCAATGGATTGAAA |
|  | Reverse:GGAAACACGACCTAACTGTTCAT |
| RP11-356O9.2 | Forward:AAACCAAAGTGGTACATGGCT |
|  | Reverse:CCCCACCAGTAACTACAAGCA |
| RP11-360P21.2 | Forward:CTGCAGTAGTGAGGGTGTTGT |
|  | Reverse:GATTCCCAAGCACCAGTCCA |
| AC012363.13 | Forward:TCCGAGCAAAGATGGGAAGC |
|  | Reverse:CATCAAGGTGTTTGGGCGTC |
| CTC-490G23.2 | Forward:CAGGCAGCAGAGACCATGAG |
|  | Reverse:AAGTCACAGGGGATGCGATG |
| RP11-1038A11.1 | Forward:CTGAATGGGAGGAGACACCG |
|  | Reverse:TCTTTCCGCGTGCTTAGGTT |
| RP11-699L21.1 | Forward:CCCTGGAAATGGCAAGGAGT |
|  | Reverse:TCGTGATAGTCCCAGAGCCA |
| LINC00113 | Forward:CCGAGTGATCGCTAAGGCAA |
|  | Reverse:GTACGCTGATTACGAGCCCA |
| GAPDH | Forward:CCGGGAAACTGTGGCGTGATGG |
|  | Reverse:AGGTGGAGGAGTGGGTGTCGCTGTT |
